# Waardenburg syndrome type II in a Chinese pedigree caused by frameshift mutation in the *SOX10* gene

**DOI:** 10.1042/BSR20193375

**Published:** 2021-06-18

**Authors:** Li Li, Jing Ma, Xiao-li He, Yuan-tao Zhou, Yu Zhang, Quan-dong Chen, Lin Zhang, Biao Ruan, Tie-Song Zhang

**Affiliations:** 1 Kunming Key Laboratory of Children Infection and Immunity, Yunnan Key Laboratory of Children’s Major Disease Research, Yunnan Medical Center for Pediatric Diseases, Yunnan Institute of Pediatrics, Kunming Children’s Hospital, Kunming 650228, Yunnan, China; 2 Department of Otolaryngology, Head and Neck Surgery, Kunming Children’s Hospital, Kunming 650228, Yunnan, China; 3 Department of Radiology, Kunming Children’s Hospital, Kunming 650228, Yunnan, China; 4 Department of Otolaryngology, First Hospital of Kunming Medical University, Kunming 650228, Yunnan, China

**Keywords:** Congenital Deafness, Iris Hypopigmentation, SOXl0 Mutation, Waardenburg Syndrome II

## Abstract

Waardenburg syndrome (WS) is a congenital hereditary disease, attributed to the most common symptoms of sensorineural deafness and iris hypopigmentation. It is also known as the hearing-pigmentation deficient syndrome. Mutations on *SOXl0* gene often lead to congenital deafness and has been shown to play an important role in the pathogenesis of WS. We investigated one family of five members, with four patients exhibiting the classic form of WS2, whose DNA samples were analyzed by the technique of Whole-exome sequencing (WES). From analysis of WES data, we found that both the mother and all three children in the family have a heterozygous mutation on the Sex Determining Region Y - Box 10 (*SOX10*) gene. The mutation was c.298_300delinsGG in exon 2 of *SOX10* (NM_006941), which leads to a frameshift of nine nucleotides, hence the amino acids (p. S100Rfs*9) are altered and the protein translation may be terminated prematurely. Further flow cytometry confirmed significant down-regulation of *SOX10* protein, which indicated the *SOX10* gene mutation was responsible for the pathogenesis of WS2 patients. In addition, we speculated that some other mutated genes might be related to disease phenotype in this family, which might also participate in promoting the progression of WS2.

## Introduction

Waardenburg syndrome (WS) was first reported in detail in 1951, a congenital hereditary disease characterized by sensorineural deafness and iris abnormalities [1]. It is also known as the auditory-pigmentary deficient syndrome. WS accounts for 2–5% of congenital deafness, and its incidence is 0.9–2.8% in deaf people [2]. According to the different clinical characteristics, WS is classified into four subtypes, namely WS1–WS4. In addition to the two most common phenotypes of sensorineural deafness and iris abnormality, WS1 is distinguished from WS2 with the W index > 1.95. The presence of limb abnormalities distinguishes WS3, and the association with Hirschsprung disease (aganglionic megacolon) is the feature of WS4 [3,4]. WS2 mainly manifests in congenital sensorineural deafness and pigment abnormalities without additional features, and symptoms of WS2 are milder than the other three.

WS is predominantly inherited as an autosomal dominant disease, the pathogenic genetic variant often occurring in an exon of a single gene. Sex Determining Region Y - Box 10 (*SOX10*) is one of them, which has been identified to contribute to the pathogenesis of WS [5–7]. Mutations in *SOX10* gene can lead to different WS phenotypes. To date, it has been found that approximately 45–55% WS4 cases and 15% WS2 cases are caused by short insertions or deletions on the *SOX10* gene [3,8].

*SOX10* is located in 22q13.1 containing five exons, in which the 3rd, 4th and 5th exons can encode proteins. *SOX10* plays an important role in the pathogenesis of WS. As it is widely expressed in early development of inner ear [9,10], a mutation on it could potentially lead to hearing deficiency. In the model of *SOX10* knockout mice, the expression levels of precursor sensory progenitor cells were decreased, causing developmental deformity of the cochlea [11,12]. *SOX10* is a key transcription factor in the development of melanocytes and intestinal ganglion cells, which can further promote the development of embryonic nerve cells and the peripheral nervous system. It either acts alone or in conjunction with other transcription factors to perform its function by binding to the promoter or enhancer of downstream target genes such as microphthalmia-associated transcription factor, regulating the melanocyte development (*MITF*), *TYR* and *TYRP*. All of these target genes are directly or indirectly involved in melanin synthesis, and their expression depends on the regulation of *SOX10* [13–16].

There were many reports of different mutations found within the *SOX10* gene in WS2 patients. Almost all of these are truncating mutations including nonsense, frameshift and one splice mutation. In the majority of cases, all or part of the transactivation domains were removed, resulting in the truncation of the protein due to a premature stop codon [17–20]. Moreover, observations predicted that most severe forms of the WS are mostly caused by mutations in the last coding exon of *SOX10*. It has been proposed that this occurs when the mutant mRNAs escape the nonsense-mediated mRNA decay (NMD) pathway [21–24].

Although many studies had been conducted on the pathogenesis of WS cases, some WS2 cases remain unexplained at the molecular level, suggesting that unknown genes may be involved or that other pathogenic mutations within the known genes have not yet been detected. This is because of the limitations of the common methods used for genotyping, and the genetic diversity in different WS families. In recent years, the number of hospitalized WS families has been increasing in China. We have already reported one case with a new mutation of *SOX10* in a previous study [25]. Here, we will describe a single WS2 family of five members, with rare situation and high genetic heterogeneity. There are four WS2 patients (the mother and her three children) with different characteristics in this family, and the father is healthy. The children inherited the mutant locus of *SOX10* from their mother with a 100% incidence. In order to describe and characterize deletions and insertions within *SOX10* in this WS2 family, we used whole-exome sequencing (WES) technique to detect the mutations. Enlightened by the phenotypic variability observed among patients with *SOX10* mutations, we also identified other genes, which may be involved in the progression of WS2.

## Materials and methods

### Subjects: all five subjects from the WS2 family were investigated

Kunming Children’s Hospital Research Ethics Committee approved the present study and informed parental consent was obtained. The proband was identified in the Otology Clinic at Kunming Children’s Hospital. Five subjects were enrolled in the present study, including the mother and three children (I-2, II-1, II-2 and II-3) of two generations and the normal father (I-1) in this family. A comprehensive medical history was carried out, and clinical evaluations including otology, ophthalmology, joints of limbs, dermatologic examinations and intelligence evaluation were taken to exclude the possibility of environmental causes. The W index was calculated by the following formula:

$$\text{X}=\frac{\left[2\text{A}-(0.2119\text{C}+3.909)\right]}{\text{C}}$$

$$\text{Y}=\frac{\left[2\text{A}-(0.249\text{B}+3.909)\right]}{\text{B}}$$

$$\text{W}=\text{X}+\text{Y}+\left(\frac{\text{A}}{\text{B}}\right).$$

Where A is the inner canthal distance, B is interpupillary distance, and C is outer canthal distance. Clinical information and DNA samples of all subjects were obtained with informed consent and in accordance with Chinese law for genetic testing. And the research has been carried out in accordance with the World Medical Association Declaration of Helsinki.

### Methods

Peripheral blood samples were collected from all members of the index family and healthy controls and processed with the TIANamp Blood DNA Kit (TianGen, Beijing, China) to extract genomic DNA for the DNA libraries. Target DNA regions of subjects were selected using the GenCap custom enrichment kit (MyGenostics Inc., Beijing, China) following the manufacturer’s protocol. Then, WES was conducted, the resultant raw image files were processed for bioinformatics analysis. Over 43000 variants were detected in the II-1, II-2 and II-3. Through the filtering of variants, the *SOX10* mutation (c.298_300delinsGG, p.S100Gfs*9) were left. It should be the ‘likely pathogenic’ mutation based on criteria using ACMG guidelines. Finally, genomic DNA from all family members was obtained for Sanger sequencing. The purified PCR products were *SOX10, POU4F3* and *COL11A1*, respectively, all of which were sequenced by Sanger Sequencing (Biosune). The sequencing results were analyzed using the DNASTAR (Madison) software.

### Flow cytometry

The expression of *SOX10, POU4F3, COL11A1, OCA2, MITF, SNAI2*, and *EDNRB* protein in peripheral blood lymphocytes of subjects was detected by flow cytometry. Antibodies were purchased from Abcam, Abclone and Bioss. The data were analyzed with Beckman Coulter DxFLEX flow cytometer and CytExpert software.

## Results

### Physical examination of five subjects

**Figure 1:**
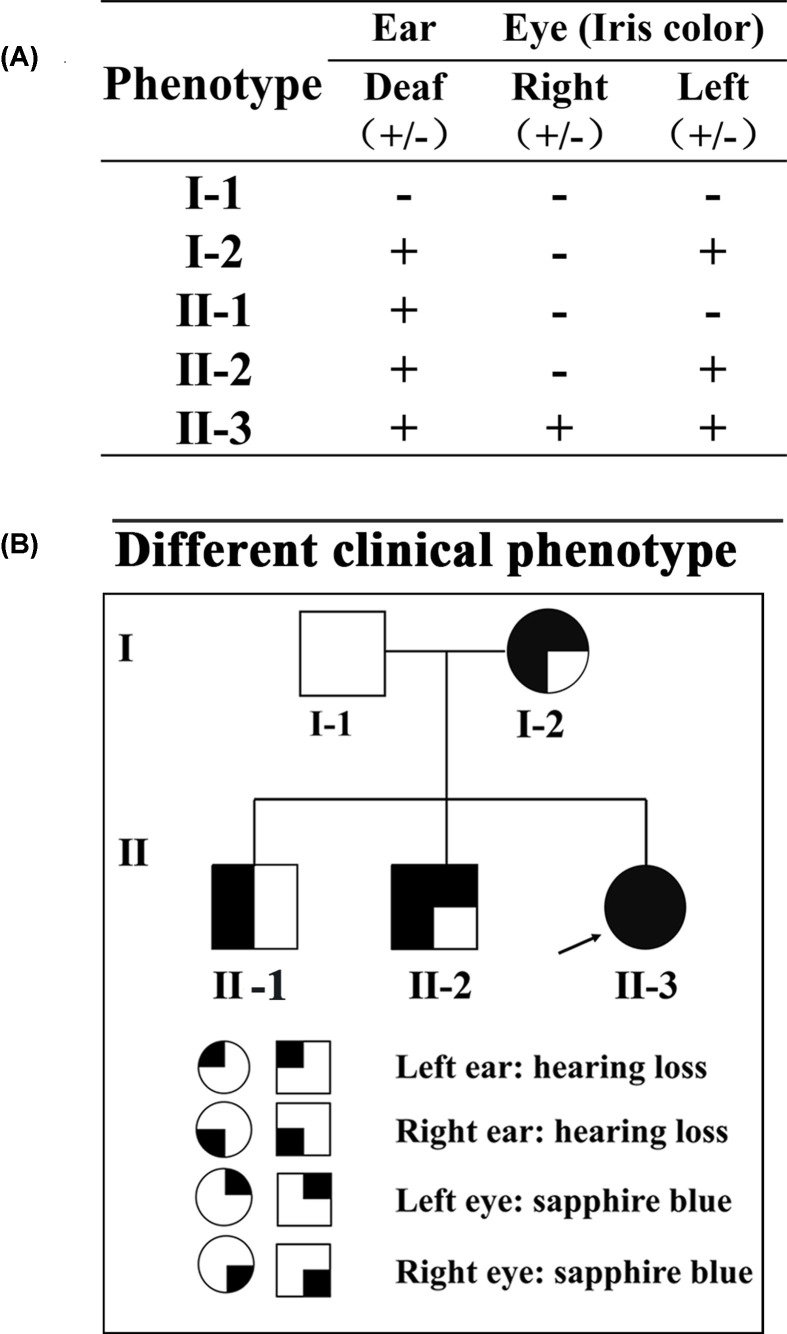
Clinical phenotypes of the five members in the WS2 family (**A**) Phenotypes of hearing status and iris color. +: abnormal; -: normal. All three children (II-1, II-2 and II-3) and the mother (I-2) exhibit hearing impairment. The father (I-1) exhibits normal hearing. (**B**) Pedigree of the family. II-3 is the proband. For the phenotype of sensorineural deafness, the mother (I-2) and three children (II-1, II-2 and II-3) are abnormal, while the father is normal. For the iris hypopigmentation, the daughter (II-3) is a binocular phenotype abnormality, the mother (I-2) and the second son (II-2) have left eye abnormality, and the eldest son (II-1) and the father (I-1) are normal.

Detailed clinical descriptions and the typical characteristics of the subjects are illustrated in Figure 1. Five subjects in this family were born at term to unrelated parents after unremarkable pregnancy and delivery. All three children had no history of ear trauma, otitis media and exposure to ototoxic drugs, no abnormalities of hair, skin or skeletal muscle. The proband (II-3), a 2-year-old girl, is the third child of non-consanguineous parents. She has iris hypopigmentation with sapphire blue eyes. Her mother (I-2, 33 years old) and second brother (II-2, 4 years old) also have iris hypopigmentation, but only their left eyes are sapphire blue, and the right eyes are brown. The iris of her eldest brother (II-1, 7 years old) is normal, with both brown eyes. The father (I-1, 36 years old) in this family is phenotypically normal, no hearing loss and iris abnormalities. Moreover, the calculated W index of four patients (I-2, II-1, II-2 and II-3) were 1.89, 1.90, 1.93, 1.92, respectively, and the four values were less than 1.95.

### Results of auditory brainstem evoked potential examination indicated severe hearing impairment in the three children of studied WS2 family

**Figure 2:**
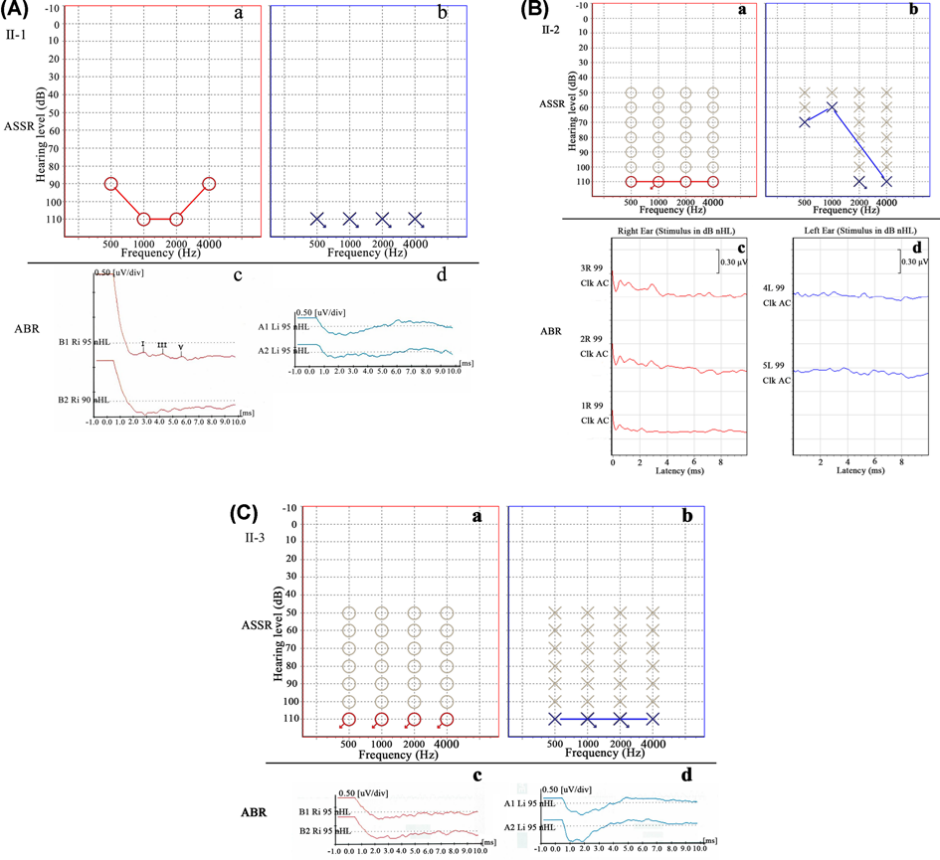
Results of auditory brainstem evoked potential examination indicated severe hearing impairment in the three children of studied WS2 family (**A**) II-1, the first son; (**B**) II-2, the second son; (**C**) II-3, the daughter. (a) and (c) in each panel show results from right ear; (b) and (d) in each panel show results from left ear; (a) and (b) present estimated pure-tone audiogram; (c) and (d) present brainstem auditory evoked potential. All three individuals exhibit severe hearing impairment (no identifiable wave at 95 dB nHL in pure-tone audiogram; no detection of binaural sound emission and double eardrum chamber curve A type in brainstem auditory evoked potential graph).

None of the four patients (I-2, II-1, II-2 and II-3) were able to communicate normally and did not respond when communicating with them. Therefore, the brainstem auditory evoked potentials were detected. As shown in Figure 2, the brainstem auditory evoked potentials of three children (II-1, II-2 and II-3) had no identifiable wave at 95 dB nHL in pure-tone audiogram and there was failure to detect binaural sound emission and double eardrum chamber curve A type in brainstem auditory evoked potential graph, which suggested that the three children (II-1, II-2 and II-3) exhibited severe bilateral hearing impairment. While the mother (I-2) developed bilateral sensorineural hearing profound deafness after a high fever at the age of 5.

### Magnetic resonance imaging examination

**Figure 3:**
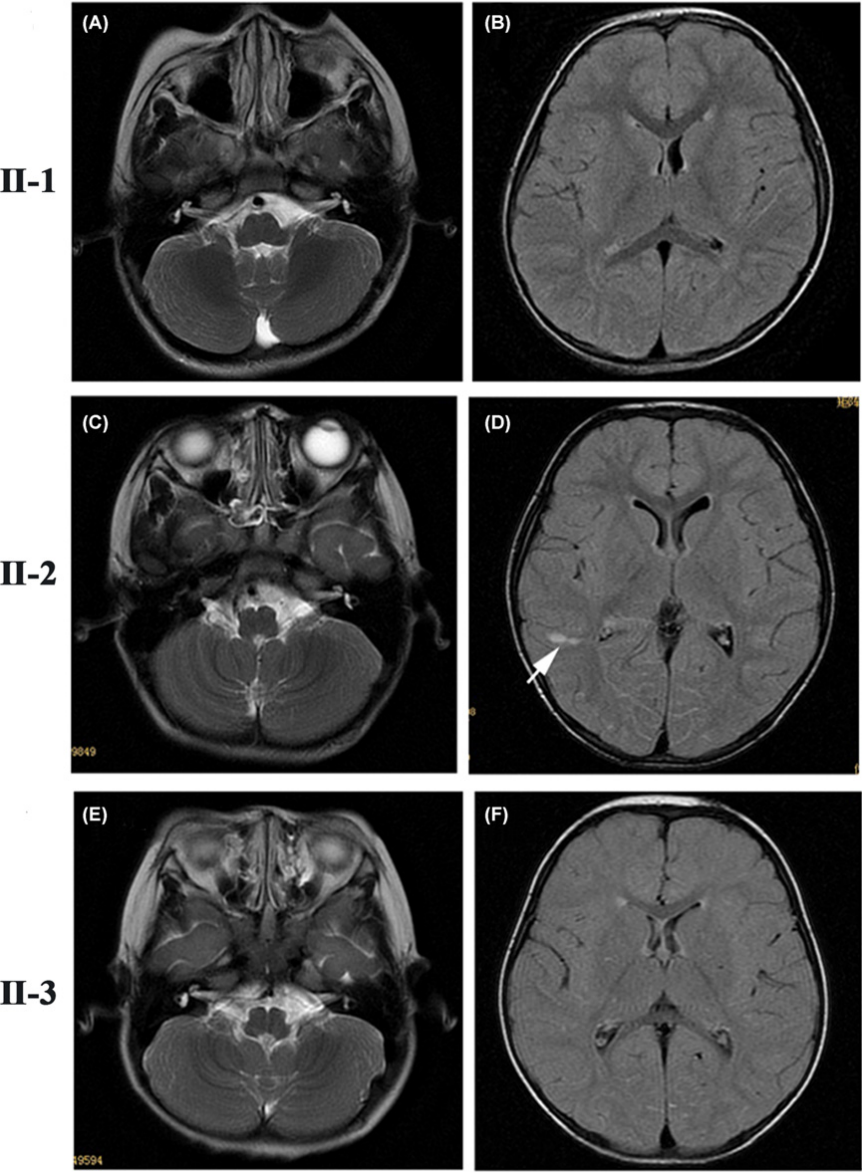
Results of MRI indicated no recognizable deficiency in the brain of the three patients of studied WS2 family The MRI images of the children (II-3, II-2 and II-1) showed some minor variations but no recognizable deficiency. (**A,B**) The MRI image of II-3 indicated that the white matter signal in the posterior angle of the bilateral ventricle and the half-oval center was altered. Clinical diagnosis suggested that it should consider as the end band. (**C,D**) The MRI result of II-2 indicated the presence of several abnormal signals such as point and flake shapes in right temporal lobe and left parietal lobe. Small-type circular cystic lesions appeared in the left hippocampus, which may suggest choroidal plexus cyst. In addition, thickening of the bilateral maxillary sinus, ethmoid sinus and sphenoid sinus mucosa was observed. (**E,F**) II-1 showed no abnormality.

Moreover, magnetic resonance imaging (MRI) was applied to determine whether the patients have brain lesions or not. As shown in Figure 3, the results indicated no recognizable deficiency in the brain of three children.

### The heterozygous mutation (c.298_300delinsGG) on *SOX10* is the pathogenic mutation in patients of the WS2 family

**Figure 4:**
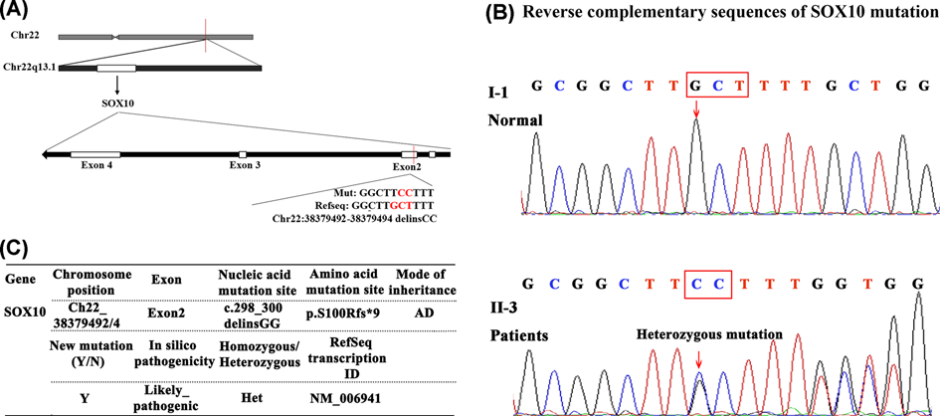
Schematic representation of the *SOX10* gene and the mutation on *SOX10* in WS2 family (**A**) *SOX10* is located on chromosome 22q13.1 including five exons, and the mutation site on *SOX 10* in this WS2 family is on exon 2. (**B**) Reverse complementary sequences of *SOX 10* mutations. Double peaks, corresponding to the sequences of the wild-type and the mutated allele, were observed beyond the position affected by the deletion. Red arrows indicate the location of potential WS2 pathogenic mutation. WT, Wild-type; MT, Mutant type. Detected sequences of subjects were aligned to the wild-type reference sequence. Double peaks at c.298_300delinsGG in exon 2 of *SOX10* (NM_006941) were found in the mother and three children indicating mutations in these four subjects (I-2, II-1 and II-2 were same to II-3, the electropherograms were not shown), the father (I-1) is normal. (**C**) Analysis of potential WS2 pathogenic mutations. Abbreviations: AD, autosomal dominant; Het, heterozygous; Y, yes.

Mutations on *SOX10* have been associated with WS2 in previous studies. The structure diagram of *SOX10* was presented in Figure 4A. As exhibited by the subjects and shown in Figure 4B, results of Sanger sequencing indicated that the likely pathogenic gene associated with this WS2 family members was *SOX10*. Except for the father (I-1), heterozygous mutations on *SOX10* gene were detected in the four affected family members (I-2, II-1, II-2 and II-3). As summarized in Figure 4C, the mutation site was found at the site c.298_300delinsGG in exon 2 of *SOX10* (NM_006941) and it caused a frameshift for nine codons and alteration of the subsequent amino acids (p. S100Rfs*9). The mutation is not positioned at a polymorphic site, and the mutation has very low incidence in the population. This is an inherited WS2 family with the *SOX10* mutation in patients.

### Expression of *SOX10, POU4F3, COL11A1, OCA2, MITF, SNAI2* and *EDNRB* in peripheral blood of the family

**Figure 5:**
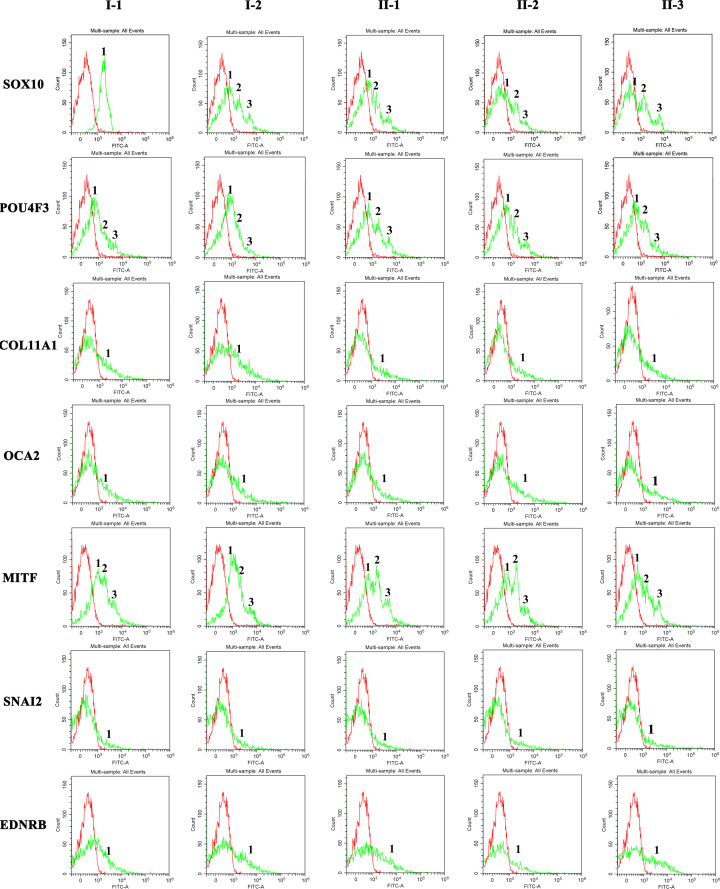
Expression of *SOX10, POU4F3, COL11A1, OCA2, MITF, SNAI2* and *EDNRB* in peripheral blood lymphocytes of the family The red peak corresponded to the blank control peak. The green peak represented the expression of seven proteins in peripheral blood of the families (from left to right: I-1, I-2, II-1, II-2, II-3).

In order to further validate the mutation genes, we performed functional protein verification. As shown in Figure 5, the red peak corresponded to the blank control peak, the green peak represented the expression of seven proteins in peripheral blood of the families. The peak values were shown in Table 1. Compared with the father, the Flow cytometry results of another four family members showed three peaks and the expression of SOX10, POU4F3 and MITF protein were down-regulated in this family, while the Flow cytometry results of *OCA2, COL11A, SNAI2* and *EDNRB* showed only one peak. In addition, the expression of four proteins in four family members (I-2, II-1, II-2, II-3) were also slightly lower than that of the father. The most obvious change was SOX10 protein expression among the seven proteins.

**Table 1:** The peak areas value of each protein expression

Gene	Family member	Peak1	Peak2	Peak3
*SOX10*	I-1	86.28%	——	——
	I-2	5.26%	24.38%	13.06%
	II-1	4.18%	24.24%	15.42%
	II-2	3.26%	19.96%	11.52%
	II-3	0.00%	21.10%	17.21%
*POU4F3*	I-1	5.54%	17.54%	9.66%
	I-2	5.18%	14.90%	7.80%
	II-1	4.50%	16.62%	8.38%
	II-2	5.20%	15.60%	9.18%
	II-3	5.08%	13.78%	8.24%
*COL11A*	I-1	20.78%	——	——
	I-2	28.66%	——	——
	II-1	15.82%	——	——
	II-2	14.24%	——	——
	II-3	16.16%	——	——
*OCA2*	I-1	19.88%	——	——
	I-2	18.48%	——	——
	II-1	15.96%	——	——
	II-2	15.32%	——	——
	II-3	15.00%	——	——
*MITF*	I-1	9.26%	23.42%	16.84%
	I-2	9.16%	22.48%	13.06%
	II-1	8.46%	21.34%	15.42%
	II-2	6.26%	20.96%	11.52%
	II-3	5.06%	16.32%	12.86%
*SNAI2*	I-1	10.20%	——	——
	I-2	8.76%	——	——
	II-1	9.12%	——	——
	II-2	8.02%	——	——
	II-3	7.44%	——	——
*EDNRB*	I-1	28.94%	——	——
	I-2	25.66%	——	——
	II-1	26.70%	——	——
	II-2	22.85%	——	——
	II-3	25.76%	——	——

## Discussion

WS2 is a complex syndrome with different clinical phenotypes in different patients. In the present study, we described a Chinese pedigree with clinical features of WS2. Four patients (I-2, II-1, II-2 and II-3) showed significant symptoms of WS2: (1) bilateral deafness (I-2, II-1, II-2 and II-3). (2) Iris hypopigmentation, including complete heterochromia iridis (II-1), sapphire blue in one eye and another one is brown (I-2, II-1). (3) Four subjects in this family all exhibited the W index < 1.95, which confirmed that they have no symptoms of dystopia canthorum. In addition, they have no history of congenital megacolon. Therefore, they were diagnosed with WS2 according to the distinguishing features of WS2.

At present, there are 61 mutations identified in the *SOX10* gene, and there are 7 and 26 types of mutations in WS2 and WS4 cases, respectively. The discovered mutation of the *SOX10* gene in the present study can be considered as a major pathogenic mutation. It was noted that the mother and the three children’s iris pigment abnormalities in the present study were not exactly the same. As shown in our results, the mother (I-2) and her two children’s (II-2 and II-3) left eye appeared sapphire blue, while the third child’s iris color was brown, which is considered normal in Chinese population. In our previous study [25], we found another WS family of one son and his parents. The son carried a novel nonsense heterozygous mutation that was found in the coding region of exon 2 in the *SOX10* gene. His parents had no mutation of relevant genes. Considering the two studies of these two WS families, we found that a new nonsense mutation had occurred in the *SOX10* gene of the proband in previous study, while the mutation of *SOX10* found in the proband of the present study was a frameshift mutation. The clinical phenotypes of both probands were sensorineural deafness and abnormal iris color. However, the genetic mutation in the previous study’s pedigree was not inherited, but *de novo*, while in this study the gene mutation of the proband and her siblings was inherited from their mother. Thus, we suggest that the same gene mutation on the *SOX10* gene with different mutated sites can result in distinct clinical phenotypes. So, further flow cytometry assay indicated the *SOX10* protein expression in the peripheral blood of the father was normal, whose iris color and hearing loss did not show abnormal symptoms. And the different expression of *SOX10* protein in the peripheral blood of the father and four patients further revealed the phenotypic heterogeneity was associated with *SOX10* gene mutation and provide a crucial understanding of the pathogenic mechanism of *SOX10* in WS2.

Whenever a complex disease occurs, it is not only due to a mutation caused by a single pathogenic gene. The presence of other related genes with mutations is highly possible, and through the interaction of these related mutant genes, the expression of protein and associated signaling pathways can be altered, resulting in abnormal development and/or metabolic disorder, and ultimately resulting in the syndrome. In addition to *SOX10*, three genes including *POU4F3, COL11A1* and *OCA2* were detected in WES data obtained in the study, which carry heterozygous point mutations in patients of this family (Supplementary Figure S1). In the present study, deficient expression of SOX10, POU4F3 and OCA2 protein was detected in four patients. According to published studies, the *POU4F3* protein was observed to play an important role in the control of cell identity in several systems. The corresponding phenotypic disorders of the mutated *POU4F3, COL11A1, OCA2* are autosomal dominant deafness type 15, Marshall syndrome (or Stickler syndrome) and eye pigment variant I, respectively [26,27]. The *COLLA1* gene encodes the type XI collagen α1 subunit, which mainly exists in the extracellular matrix of cartilage and plays an important role in the assembly of cartilage collagen fibers. Its mutation can lead to Marshall syndrome, Stickler Syndrome II, osteoarthritis etc. with the clinical phenotype of hearing loss and the typical skeletal and facial manifestations [28,29]. The *OCA2* protein plays a central role in melanosome biogenesis, its mutation resulting in an autosomal recessive disorder characterized by hypopigmentation of the skin, hair and eyes, accompanied with ophthalmologic abnormalities. *OCA2* is also a strong determinant of the eumelanin content in melanocytes. *OCA2* can regulate the pH within melanosomes and affect melanosome maturation. It is the major determinant of brown and/or blue eye color [30,31]. *SOX10* mutations can lead to abnormal development of the inner ear, while in this process the *COL11A1* mutation exacerbates the anomaly due to the development disorder of cartilage. This is also likely to be one of the key causes of abnormal development of inner ear. On the other hand, defects in EDNRB and SNAI2 proteins have also been detected. Because of the small percentage of EDNRB and SNAI2 mutations among WS2 patients, there are few reports on whether the EDNRB and SNAI2 mutation is associated with the pathogenesis of WS2 [32,33]. The relationship and mechanism between the two genes and WS2 pathogenesis needs more researches to clarify. Therefore, we predict that *SOX10* was the major pathogenic gene in this family, and other genes might also contribute to the disease phenotypes.

*SOX10* mutation can affect the expression of its upstream and downstream genes, thereby resulting in differences in cell phenotypes. Being a key transcription factor in the neural crest cell (NCC) migration and differentiation processes, *SOX10* can function independently or in concert with other transcription factors by binding to the promoter or enhancer of the target gene [15]. In order to further identify the relationship between *SOX10* and other genes that might contribute to WS2 progression, we searched the gene regulatory networks of these mutated genes through Transcriptional Regulatory Relationships Unraveled by Sentence-based Text mining (TRRUST: http://www.grnpedia.org/trrust), a reference database of human transcriptional regulatory interactions. As shown in Supplementary Figure S2, we searched several *SOX10*-related genes such as the *CEBPZ* (CCAAT-Box binding transcription factor), which regulates the expression of its target gene *SOX9*, and *SOX9* is known as a target gene of *SOX10* [34,35]. Thus, as the upstream transcriptional regulatory factor of the *SOX9* gene, *CEBPZ* can also affect the expression of *SOX10*. Moreover, *SOX10* can further modulate the expression of the *MITF* [36], its downstream target gene, which in turn can also promote the activation of *OCA2*, which is the target gene of *MITF* [37,38]. MITF was also detected with defects in its protein expression, its mutation may also be closely related to the pathogenic of WS2. In addition, *CEBPZ* may also control the expression of *COL11A1* [39].

According to the gene regulatory networks found through TRRUST, we assumed that the *SOX10* gene mutation could influence expression of its upstream genes such as *CEBPZ* and *SOX9*, or its target gene such as *MITF3* [32,40]. Some reports have described similarities between the functions of *CEBPZ* and *MITF3*. Both of them play an important role in the transcriptional regulation of cell differentiation and proliferation, such as in neural crest-derived melanin cells, mast stem cells, osteoclasts and cells from the retinal pigment epithelium derived from the optic cup [6,40]. We did not find any mutations in *SOX9, CEBPZ* and *MITF* in our WES data of this family. We speculate that although no mutation was detected in these genes, their expression might be suppressed or terminated due to the mutation in downstream gene *SOX10* causing their corresponding functions to be weakened or even lost.

In conclusion, as a key transcription factor in NCC migration and differentiation, *SOX10* can function independently or in concert with other transcription factors by combining with the promoter or enhancer of the target gene. Different types of mutations in the *SOX10* gene might affect the clinical phenotypes through different signal pathways in WS. Since the clinical phenotypes of the family of the present study were more severe than the ones of other WS patients and it has significant genetic characteristics. The research conducted on the pathogenesis of this family can help us find more pathogenic molecular mechanisms associated with deafness and abnormal pigmentation, and to further diagnose and discover new mutations in the WS-related genes. It can also provide a good basis for our disease diagnosis and treatment. Moreover, the follow-up mechanism and more cases research will be continued to provide more valuable supporting data to elucidate the development mechanism of WS pathogenesis, which will aid in steps toward breakthrough discoveries for improved and specialized treatment of WS patients.

## Supplementary Material

Supplementary Figures S1-S2

## Data Availability

The data used to support the findings of the present study are included within the article and supplementary files.

## Abbreviations

*CEBPZ*: CCAAT-box binding transcription factor
*MITF*: microphthalmia-associated transcription factor, regulating the melanocyte development
NCC: neural crest cell
*SOX10*: sex determining region Y - box 10
TRRUST: Transcriptional Regulatory Relationships Unraveled by Sentence-based Text mining
WES: whole-exome sequencing
WS: Waardenburg syndrome
