# Supplementary material for: Waardenburg syndrome type II in a Chinese pedigree caused by frameshift mutation in the SOX10 gene
Source: Biosci Rep. 2021 Jun 18;41(6):BSR20193375. doi: 10.1042/BSR20193375 (PMC8217986; doi:10.1042/BSR20193375)
Supplement: Supplementary Figures S1-S2 [file BSR-2019-3375_supp.pdf]

A

Genes	POU4F3	COL11A1	OCA2
Chromosome position	Chr5_ 145719613	Chr1_ 103453270	Chr15_ 28230211
Exon	Exon2	Exon30	Exon13
Nucleic acid mutation sites	c.623T>C	c.2457A>T	c.1363A>G
Amino acid mutation sites	p.V208A	p.R819S	p.R455G
Mode of inheritance	AD	AD	AR
New mutation (Y/N)	Y	Y	N
In silico pathogenicity analysis	Uncertain	Uncertain	Likely_ pathogenic
Homozygous/ Heterozygous	Het	Het	Het
RefSeq transcript ID	NM_002700	NM_080629	NM_000275

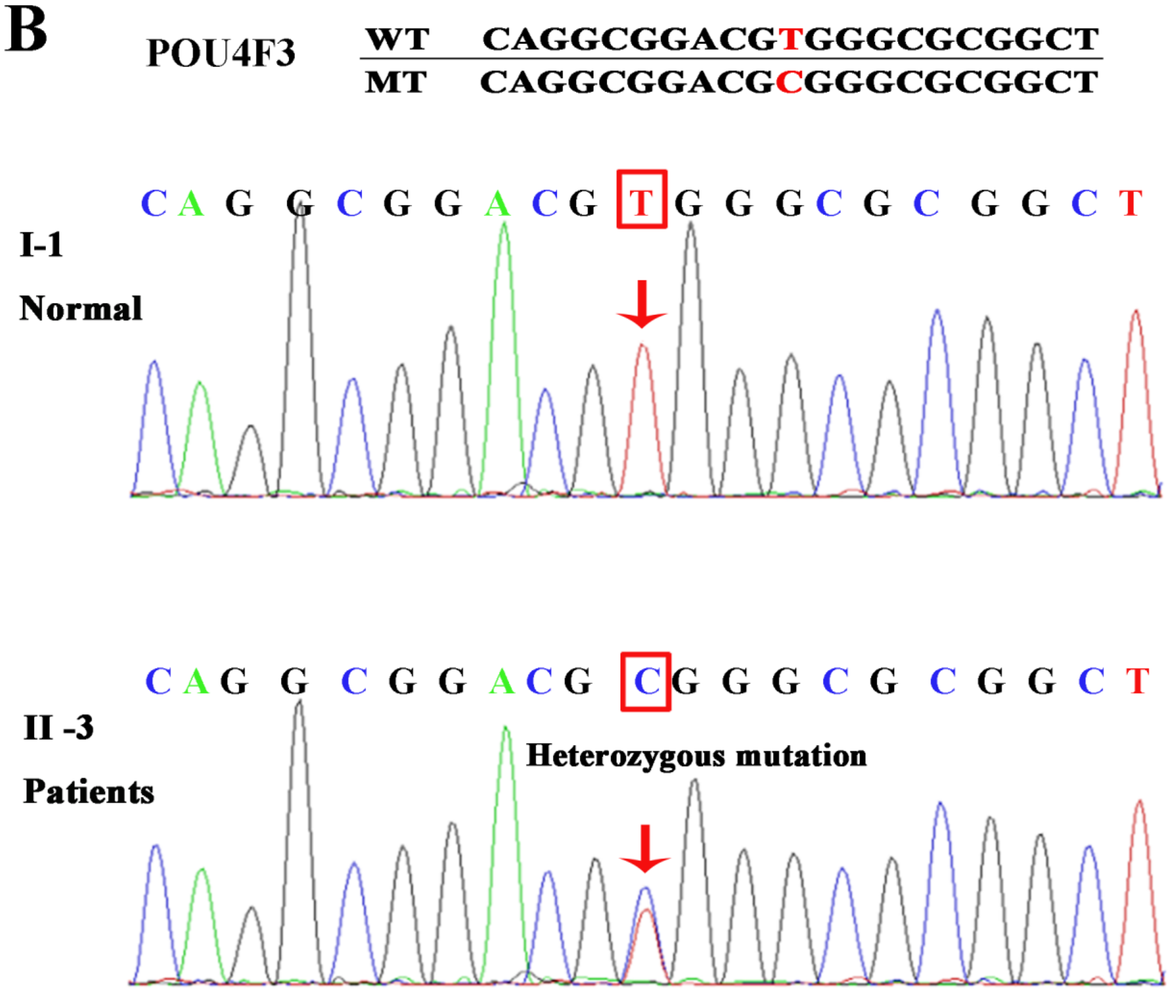
B
POU4F3
WT CAGGCGGACGTGGGCGCGGCT
MT CAGGCGGACGCGGGCGCGGCT
C A G G C G G A C G T G G G C G C G G C T
I-1
Normal
C A G G C G G A C G C G G G C G C G G C T
II -3
Patients
Heterozygous mutation

C **Reverse complementary sequences of COL11A1 mutation**

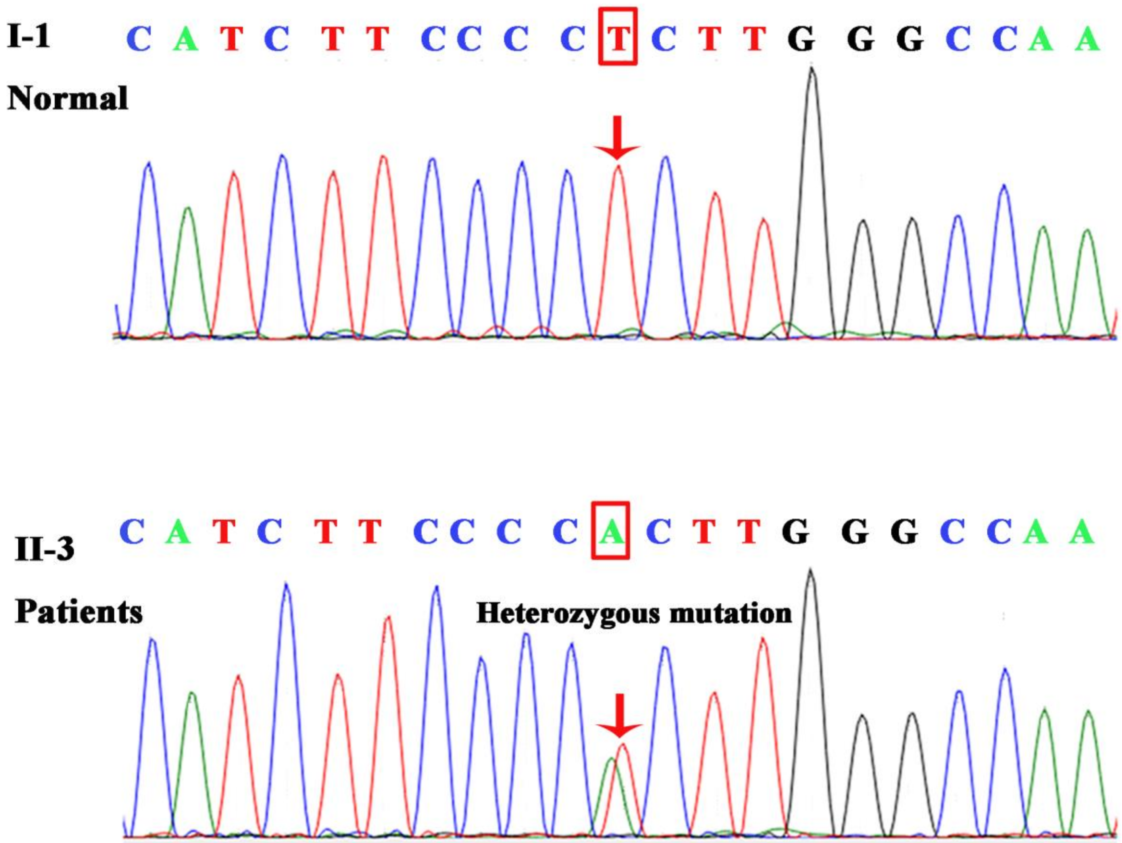

D Reverse complementary sequences of OCA2 mutation

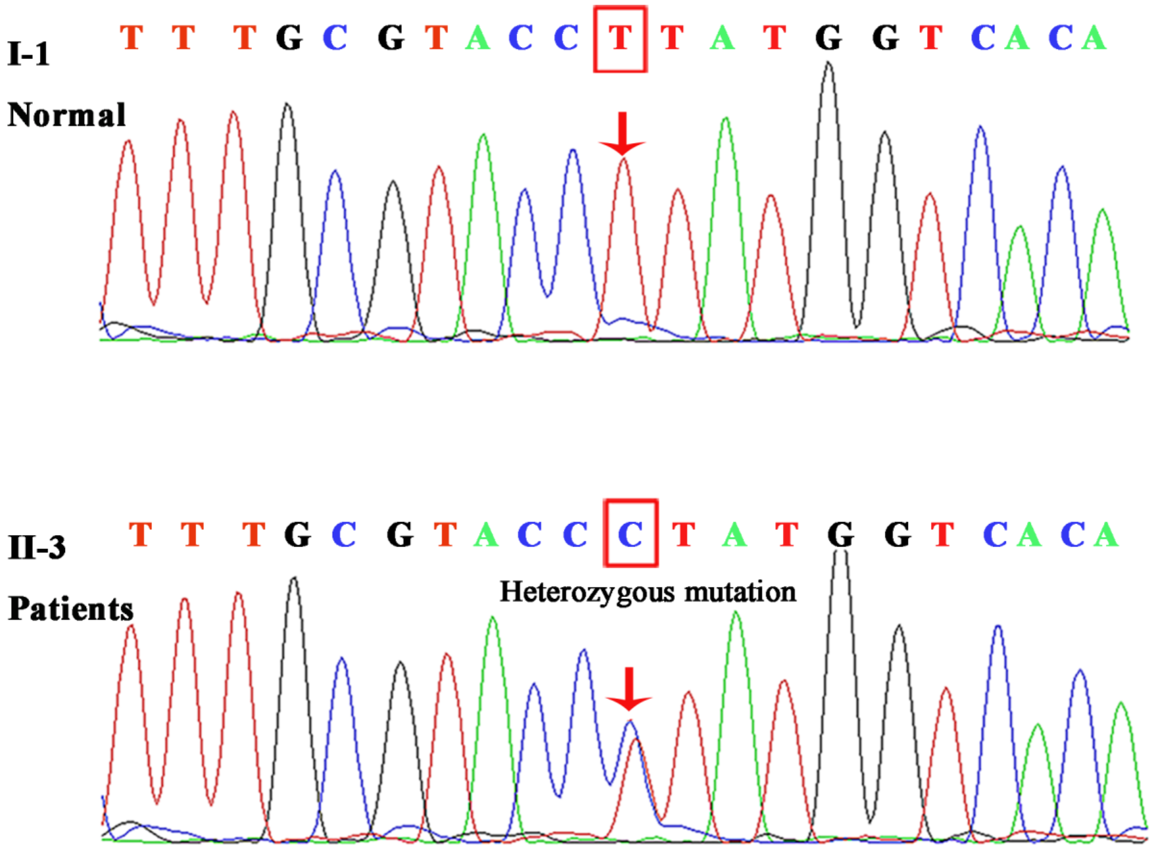

Figure S1. Mutations detected in three genes were possibly related to the symptom of WS2. Results of Sanger sequencing indicated the mutation on *POU4F3* gene [NM_002700 c.623T>C], *COL11A1* gene [NM_080629 c.2457A>T], and *OCA2* gene [NM_000275 c.1363A>G]. The mutations also have been registered in dbSNP database as rs774435223, rs367824632, and rs200764804. A. Analysis of potential WS2 pathogenic mutations. AD: autosomal dominant; AR: autosomal recessive; Y: yes; N: no; Het: heterozygous. **B.** Double peaks at c.623T>C in exon 2 of *POU4F3* (NM_002700) were found in the daughter, the first son and the mother indicating mutations in these three subjects (I-2 and II-1 were same to II-3, the electropherograms were not shown), the father and the second son (I-2 and II-2) is normal (II-2 were same to I-1, the electropherograms was not shown). **C.** Reverse complementary sequences of *COL11A1* mutation. Double peaks at c.2457A>T in exon 30 of *COL11A1* (NM_080629) were found in the daughter and the mother (II-3 and I-2) indicating mutations in these two subjects, the father and the two sons (I-1 II-1 and II-2) are normal. **D.** Reverse complementary sequences of *OCA2* mutations. Double peaks at c.136A>G in exon 13 of *OCA2* (NM_000275) were found in the mother, second son and the daughter (I-2, II-2 and II-3) indicating mutations in these three subjects, the father and the eldest son (I-1 and II-1) are normal.

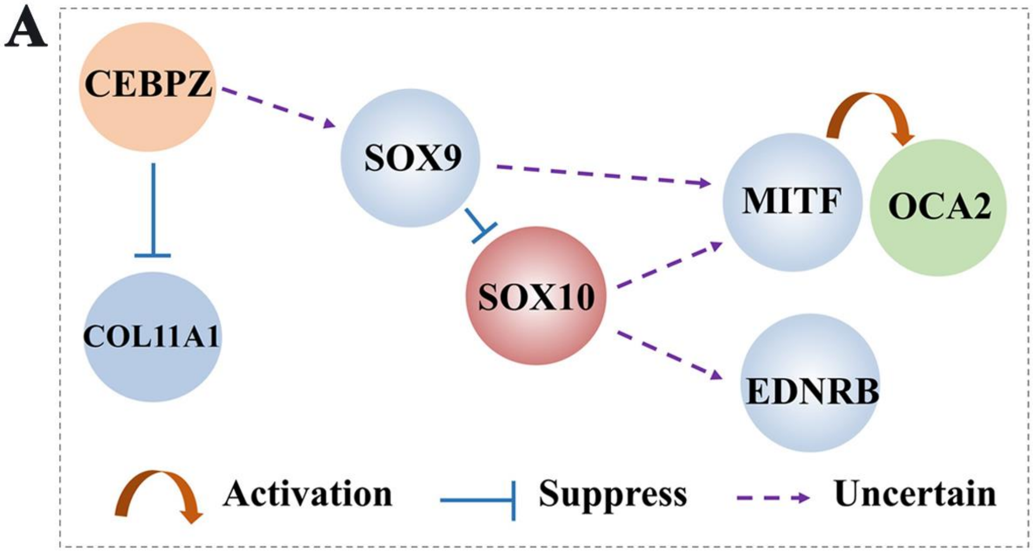

B

	Transcription Factor (TF)	Target gene	Regulation	Reference (PMID)
1	CEBPZ	SOX9	Uncertain	12805396
2	CEBPZ	COL11A1	Suppress	15908194
3	SOX9	MITF	Uncertain	17702866
4	SOX10	MITF	Uncertain	19026785
5	MITF	OCA2	Activation	22234890
6	SOX9	SOX10	Suppress	15896776
7	SOX10	EDNRB	Uncertain	16623715

Figure S2. Transcription Factors (TFs)' interactions of *SOX10* related genes. A. *SOX10* related genes, such as *CEBPZ*, which regulates the expression of its target gene *SOX9*, which in turn, is also a target gene of *SOX10*. As the upstream transcriptional regulatory factor of the *SOX9* gene, *CEBPZ* can also affect the expression of *SOX10*. Moreover, *SOX10* can further modulate the expression of the *MITF*, its downstream target gene, which in turn can also promote the activation of *OCA2*, which is the target gene of *MITF*. In addition, *CEBPZ* may also control the expression of *COL11A1*. **B.** Regulations between transcription factors and target genes.
